# Targeting S100A8/A9: a molecular gateway to oral inflammation, microglial activation and neuroinflammation

**DOI:** 10.3389/fimmu.2026.1888031

**Published:** 2026-08-24

**Authors:** Jonaid Ahmad Malik, Tooba Qamar, Abrar H. Qadri, Iqball Faheem

**Affiliations:** 1 Department of Biomedical Engineering, Indian Institute of Technology, Ropar, India; 2 Department of Clinical Immunology and Rheumatology, Sanjay Gandhi Postgraduate Institute of Medical Sciences (SGPGIMS), Lucknow, Uttar Pradesh, India; 3 Department of Biochemistry, School of Life Sciences, University of Hyderabad, Hyderabad, TG, India; 4 Department of Microbiology and Cell Biology, Indian Institute of Science, Bangalore, India

**Keywords:** microglia activation, neuroimmune communication, neuroinflammation, NLRP3 inflammasome, protein therapeutics, rage, S100A8/A9, salivary biomarkers

## Abstract

S100A8 and S100A9 are critical members of the S100 protein family with diverse immunoregulatory functions, serving as important mediators of neuroimmune communication and key links between inflammation, microglial activation, and neurodegenerative processes. S100A8 and S100A9 are abundantly expressed in neutrophils, monocytes, and other immune cells and are rapidly released during inflammatory responses. Once extracellular, S100A8/A9 interacts with cell-surface pattern-recognition receptors, including TLR4 and RAGE, triggering downstream NF-κB and MAPK signaling cascades that drive pro-inflammatory cytokine production. S100A8/A9 has also been implicated in activating the intracellular NLRP3 inflammasome sensor/scaffold, a pro-inflammatory response associated with neuroinflammation. Growing evidence indicates that dysregulated S100A8/A9 expression contributes to both central and peripheral inflammatory pathologies and is elevated across multiple disease states. This review provides a comprehensive analysis of the S100A8/A9 signaling axis, examining its structural and functional roles in regulating microglial activation and neuroimmune crosstalk. It further explores the role of S100A8/A9 signaling in the pathogenesis of diverse inflammatory and neurodegenerative disorders, highlighting the underlying molecular mechanisms. By integrating current knowledge, this review highlights the therapeutic potential of targeting the S100A8/A9 to modulate neuroinflammation and facilitate the development of more effective interventions for neurodegenerative diseases.

## Introduction

S100A8/A9, also known as calprotectin, is a member of the S100 family of calcium-binding proteins. It has attracted significant attention as an important alarmin that regulates inflammatory responses upon its release from activated neutrophils and monocytes (1). These proteins are constitutively expressed in neutrophils and monocytes and function as calcium sensors involved in cytoskeletal dynamics and arachidonic acid metabolism. Under inflammatory conditions, S100A8/A9 is actively secreted and plays an important role in regulating inflammation by promoting leukocyte recruitment and stimulating cytokine production (2). The involvement of S100A8 and S100A9 in inflammation was first identified nearly two decades ago, but their significance in rheumatic disease pathogenesis has only recently gained prominence. Emerging evidence further implicates S100A8/A9 in cancer progression, obesity, neurodegeneration, and atherosclerotic plaque formation (3).

S100A8/A9 levels were markedly elevated in patients with typhoid fever, correlated with fever duration, and showed direct antimicrobial effects against *S. Typhi* and *S. Typhimurium in vitro* (4). S100A8/A9 is highly expressed in the cytoplasm of neutrophils and epithelial cells and functions both within cells and in tissues during infection and inflammation. In epithelial cells, it helps inhibit the growth of various oral and intestinal pathogens, including *Porphyromonas gingivalis*, *Salmonella enterica* serovar Typhimurium, and *Listeria monocytogenes* (5). S100A8/A9, a calcium-binding hetero-oligomer composed of S100A8 and S100A9 subunits, possesses antimicrobial activity by chelating transition metals, such as iron, manganese, zinc, and nickel, which are essential cofactors for numerous bacterial enzymes (5). In addition, the cellular localization and expression patterns of key S100 family members especially S100A8 (calgranulin A), and S100A9 (calgranulin B), have been investigated across various oral lesions. The findings revealed that S100A8 and S100A9 transcripts were markedly reduced in malignant tissues, suggesting their potential as molecular markers for assessing malignancy risk in oral pathology (6).

Accumulating evidence implicates the role of S100A8/A9 complex in the pathogenesis of multiple neurodegenerative disorders, where it contributes to neuroinflammation, cognitive decline, and disease progression (7–9). Elevated levels of S100A8/A9 have been consistently detected in the blood and cerebrospinal fluid (CSF) of patients with encephalopathy, further supporting its association with central nervous system (CNS) pathology (9–11). Consequently, S100A8/A9 has emerged as a promising therapeutic target for a range of neurological and systemic disorders, including sepsis, Alzheimer’s disease (AD), stroke, and systemic lupus erythematosus (SLE). Neuroinflammation is a central pathological feature of many CNS disorders and is strongly associated with impairments across multiple cognitive domains (9). As key mediators of neuroimmune communication, S100A8 and S100A9 regulate microglial activation and inflammatory signaling pathways. Dysregulation of these proteins disrupts neuroimmune homeostasis, driving a complex interplay between immune signaling, chronic inflammation, and microglial dysfunction that ultimately accelerates neurodegeneration and cognitive impairment. Despite increasing recognition of these mechanisms, effective therapeutic strategies for treating cognitive dysfunction remain limited, underscoring the need to explore S100A8/A9-targeted interventions as a potential avenue for mitigating neuroinflammation and improving clinical outcomes.

The emphasis on S100A8/A9 as the molecular link in protein-mediated neuroimmune communication between oral inflammation and microglial activation is well supported by elevated levels of S100A8/A9 in the oral cavity at the onset of periodontitis (12). In this condition, the heterodimer is oversecreted by neutrophils and macrophages in response to dysbiosis caused by *Porphyromonas gingivalis* and *Fusobacterium nucleatum*, leading to tissue damage and enhanced inflammation through toll like receptor 4 (TLR4)/(Nuclear factor kappa-light-chain-enhancer of activated B cells) NF-κB-mediated interleukin-6 (IL-6) production by gingival fibroblasts (13). This high oral bioavailability guarantees high systemic bioavailability, as indicated by the correlation between saliva and blood. Furthermore, by directly binding to TLR4/RAGE (Receptor for Advanced Glycation End Products) on BV-2 cells and primary cultures, S100A8/A9 exhibits high receptor fidelity in microglia (14). This triggers the MyD88/Interleukin-1 Receptor-Associated Kinase 1 (IRAK-1)/NF-κB and mitogen-activated protein kinase (MAPK)/p38 cascades, driving dose-dependent M1 polarization, strong cytokine release, and neuronal apoptosis, effects that are blocked by RAGE antibodies or TLR4 inhibitors (9). Elevated periodontal S100A8/A9 drives Aβ/tau pathology and persistent microglial priming in *P. gingivalis* periodontitis models, whereas inhibitors such as paquinimod reduce CNS inflammation (15). These findings strengthen S100A8/A9 as a key therapeutic target in the oral-brain inflammatory axis. In this review, we aim to summarize and discuss current studies on S100A8/A9 as a key therapeutic target, explaining its structure, exploring its intricate regulatory network, and discussing its implications for oral and neurological health.

## Structural properties of S100A8/A9

**Figure 1:**
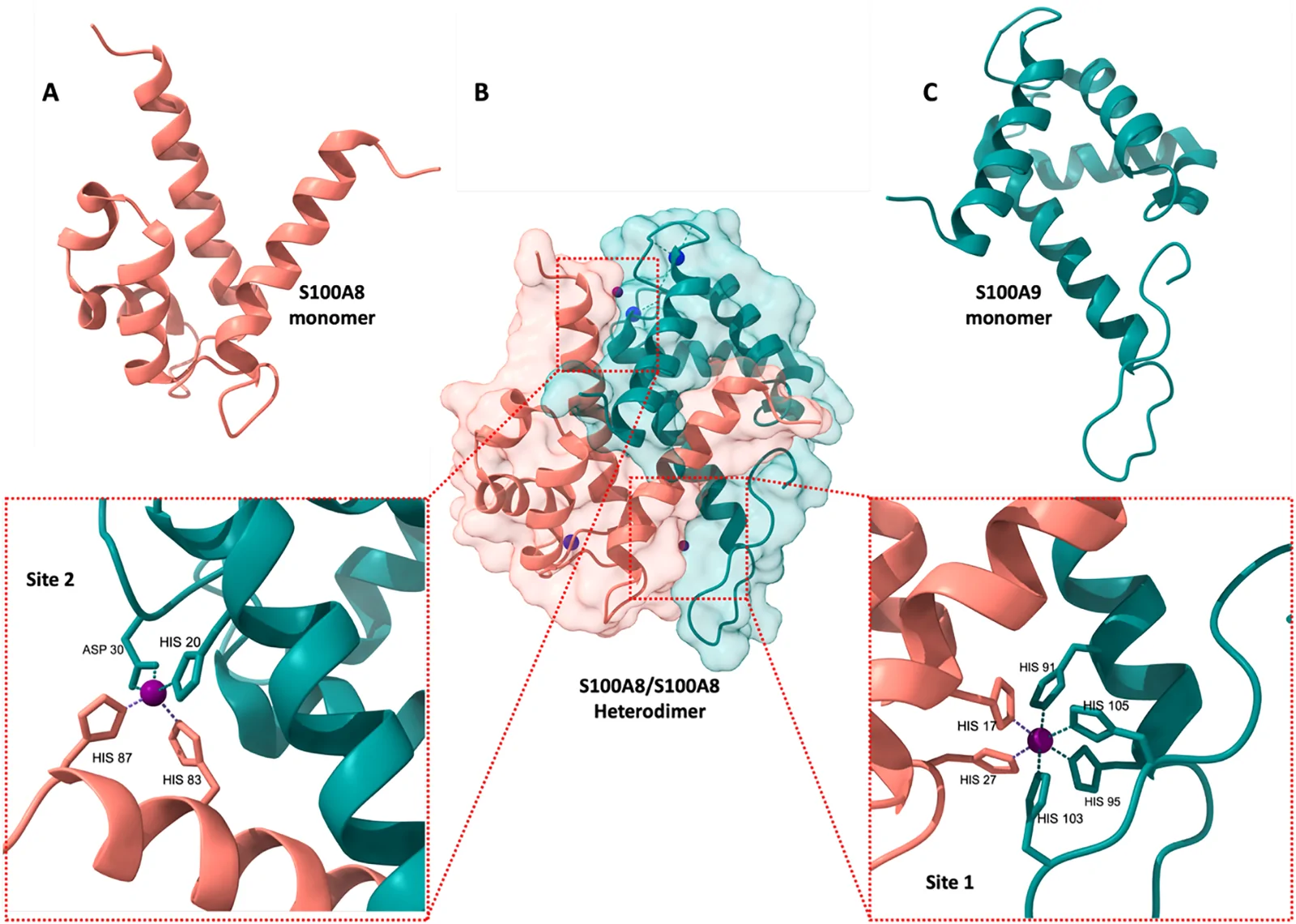
Structure of S100A8/A9. **(A)** S100A8 monomer (salmon). **(B)** S100A8/A9 (S100A8/S100A9 heterodimer) containing two Mn^2+^ ions (purple) bound at site 1 and site 2, shown as zoomed sections. The Ca^2+^ ions bound to the dimer are represented in blue. **(C)** The S100A9 monomer is shown in teal. (Figure generated from crystal structure PDB: 4GGF).

The S100 protein family comprises small (~10 kDa) calcium-binding proteins named for their solubility in fully saturated ammonium sulfate solutions. Among these, S100A8 and S100A9 consist of 93 and 113 amino acids, respectively, with S100A9 also occurring as a truncated 110 amino acid isoform. These proteins assemble predominantly into stable heterodimers, although homodimer formation has also been observed under both *in vitro* and *in vivo* conditions. Structurally, each protein possesses a characteristic helix–loop–helix EF-hand motif rich in charged residues, a structural hallmark of the S100 family that confers a strong binding affinity for divalent cations such as Ca^2+^, Mn^2+^, and Zn^2+^ (16–18) (**Figure 1**). S100A8 and S100A9 preferentially associate to form the heterodimeric complex known as S100A8/A9, which exhibits strong affinity for Mn^2+^. At the molecular level, Mn^2+^ coordination by S100A8/A9 is mediated by six histidine residues at site 1: 4 forming a canonical tetrahedral metal-binding site, complemented by two additional histidine residues contributed by the C-terminal tail of S100A9 (19). This hexa-histidine coordination environment is a distinctive feature that enables high-affinity Mn^2+^ binding. Site 2 is proposed to bind Zn^2+^ preferentially and Mn^2+^ with low affinity (20, 21). Metal binding is known to influence the structural and functional properties of S100A8/A9. For instance, Mn^2+^ binding promotes ordering of the S100A9 C-terminal tail, suggesting that Mn-bound S100A8/A9 may exhibit distinct immunomodulatory activities compared to its Zn-bound or metal-free forms (22). Additionally, Ca^2+^ binding to the EF-hand domains enhances Mn^2+^ affinity at the primary histidine-rich site, shifting it from the low micromolar to the mid-nanomolar range. Although S100A8/A9 displays an overall preference for Zn^2+^ over Mn^2+^, the specificity of Mn^2+^ for the histidine-rich site enables the formation of heterometallic complexes, in which Zn^2+^ and Mn^2+^ occupy distinct binding sites within the same protein complex (22).

Due to its release at inflammatory sites, particularly at the site of infection, S100A8/A9 serves as a primary component of host defense against invading pathogens (23). It has been proposed that eukaryotic systems may have evolved antimicrobial strategies that exploit the preferential utilization of Mn^2+^ by pathogens in essential enzymatic processes (22, 24). The antibacterial effect is mediated through metal ion-binding capacity; specifically, S100A8/A9 sequesters metal ions, thereby depriving pathogens of these essential cofactors and inhibiting their growth (20, 25). Beyond its antimicrobial function, S100A8/A9 also participates in immune signaling, acting as a ligand for TLR4 and the receptor for advanced glycation end products and serving as a chemoattractant for macrophages (22, 26, 27).

### S100A8/A9–controlled signaling axis: a central driver of inflammation and neuroinflammation

S100A8/A9 functions as an endogenous damage-associated molecular pattern (DAMP) that is rapidly released following tissue injury and inflammatory stress. Extracellular S100A8/A9 primarily binds to TLR4, initiating MyD88-dependent signaling that activates downstream inflammatory pathways, including NF-κB, MAPK, and the NLR family pyrin domain containing 3 (NLRP3) inflammasome (1, 26, 28). Under physiological conditions, S100A8/A9 expression is tightly regulated by lineage-specific transcription factors, such as PU.1 and C/EBPα/β, as well as epigenetic mechanisms that maintain low basal expression. During infection or tissue damage, microbial PAMPs and endogenous DAMPs activate the TLR4/MyD88/NF-κB pathway, resulting in robust induction of S100A8/A9 expression and amplification of inflammatory responses (2, 29). Beyond its own induction, S100A9 directly activates TLR4 signaling to stimulate ROS-dependent production of IL-6 and IL-8, demonstrating its role as a potent pro-inflammatory mediator (30). Although S100A8/A9 is widely recognized as a pro-inflammatory mediator, its biological activity is context-dependent and varies according to the cellular source, tissue microenvironment, and stage of disease progression (31). During influenza A virus infection, extracellular S100A9 functions as a non-PAMP activator of TLR4 signaling, promoting inflammation, cell death, and viral pathogenesis through the TLR4-MyD88-NLRP3 pathway (32). In the CNS, activation of TLR4 by S100A8/A9 promotes microglial activation, neuroinflammation, neuronal apoptosis, and cognitive dysfunction, thereby linking peripheral inflammatory responses with neurodegenerative disease progression (33, 34) (**Table 1**).

**Table 1:** Role of S100 proteins in microglial activation and neuroimmune signaling.

S100 protein(s)	Cell/Model	Stimulus	Signaling pathway	Effect	References
S100A8/A9	BV2 microglia	–	NF-κB	Drives M1 microglial activation, boosts cytokines/chemokines, induces OPC apoptosis, exacerbates MS neuroinflammation	(15)
S100A8	Neurons & microglia	Hypoxia	ERK/JNK, NLRP3 inflammasome	Mediates microglia-neuron crosstalk, induces pro-inflammatory cytokines, COX-2 expression, and neuronal apoptosis	(33)
S100A8/A9	Microglia	–	TLR4, RAGE → ERK/JNK → NF-κB	Promotes TNFα and IL6 secretion; therapeutic target for neuroinflammation	(14)
S100A8	Glioma cells	–	TLR4 → NF-κB/IL-10	Promotes proliferation, migration, invasion; drives microglial M2 polarization; correlates with poor prognosis	(35)
S100A8	Microglia	Migraine model	–	Contributes to pain and neuroinflammation; inhibition reduces activation, cytokine release, and pain	(36)
S100A9	Hippocampus/microglia	Sepsis	–	Promotes M1 polarization, memory impairments; inhibition shifts microglia to M2, improves survival and cognition	(37)
S100A8/A9	Microglia	Tauopathy/aging	–	High expression in hippocampal microglia correlates with cognitive impairment; detected in human AD and aged brains	(38)
S100A8/A9	Neutrophils/spinal cord	Carrageenan-induced peripheral inflammation	–	Upregulation in neutrophils, endothelial activation, and potential CNS release may signal peripheral tissue damage	(39)
S100A8/A9	Neutrophils & macrophages	–	Post-translational & oxidative modifications	Influence both pro- and anti-inflammatory functions; modifications act as regulatory switches	(40)
S100A8/A9	Neutrophils	ATP stimulation	P2Y purinergic receptors	Do not buffer Ca2+/Zn2+ generally; ATP triggers stronger intracellular Ca2+ in S100A9-deficient cells	(41)
S100A8/A9	Microglia	Tissue injury	Oxidative & post-translational modifications	Modulate immune functions; reversible modifications limit inflammation, modulate mast cells, leukocyte recruitment, and NO-mediated vascular responses	(42)
S100A8/A9	CNS	CNS diseases (AD, PD, MS, stroke)	–	Promote leukocyte recruitment and cytokine release; biomarkers and therapeutic predictors	(43)
S100A8	Neurons & microglia	Hypoxia	TLR4 → ERK/JNK, NLRP3 inflammasome	Promotes neuroinflammation and neuronal apoptosis; regulates COX-2 in microglia; therapeutic target	(33)
S100A9	BV2 microglia	AD	TLR4/7 → TNFα, IL6	Low dose: promotes proliferation/migration; high dose: cytotoxic; Aβ 1–40 rescues cells	(34)
S100A8/A9	CNS/systemic	Cognitive decline	–	Affects memory, attention, learning, emotion; potential diagnostic marker and therapeutic target	(9)
S100A9	CNS	AD & PD	Amyloid plaque formation	Drives neuroinflammation and neurotoxicity; targeting S100A9 (e.g., hydroxytyrosol) may reduce aggregation and inflammation	(44, 45)

NF-κB represents one of the principal downstream effectors of S100A8/A9 signaling. Following TLR4 activation, MyD88-dependent recruitment of interleukin-1 receptor-associated kinases (IRAKs) and tumor necrosis factor Receptor-Associated Factor 6 (TRAF6) leads to degradation of nuclear factor of kappa light polypeptide gene enhancer in B-cells inhibitor alpha (IκBα) and nuclear translocation of NF-κB, initiating transcription of numerous inflammatory mediators, including Tumor Necrosis Factor alpha (TNF-α), IL-1β, IL-6, and multiple chemokines (46). This signaling cascade establishes a positive inflammatory feedback loop that perpetuates immune cell recruitment and chronic inflammation. Within the CNS, persistent activation of NF-κB by S100A8/A9 promotes microglial polarization toward the pro-inflammatory M1 phenotype, resulting in enhanced cytokine production, oxidative stress, and neuronal injury (47, 48). Elevated S100A8/A9 levels in multiple sclerosis patients drive NF-κB-dependent microglial activation, which contributes to oligodendrocyte precursor cell apoptosis and demyelination. Pharmacological inhibition of NF-κB partially reverses these pathological changes, highlighting the importance of this pathway in S100A8/A9-mediated neuroinflammation (15, 48).

**Figure 2:**
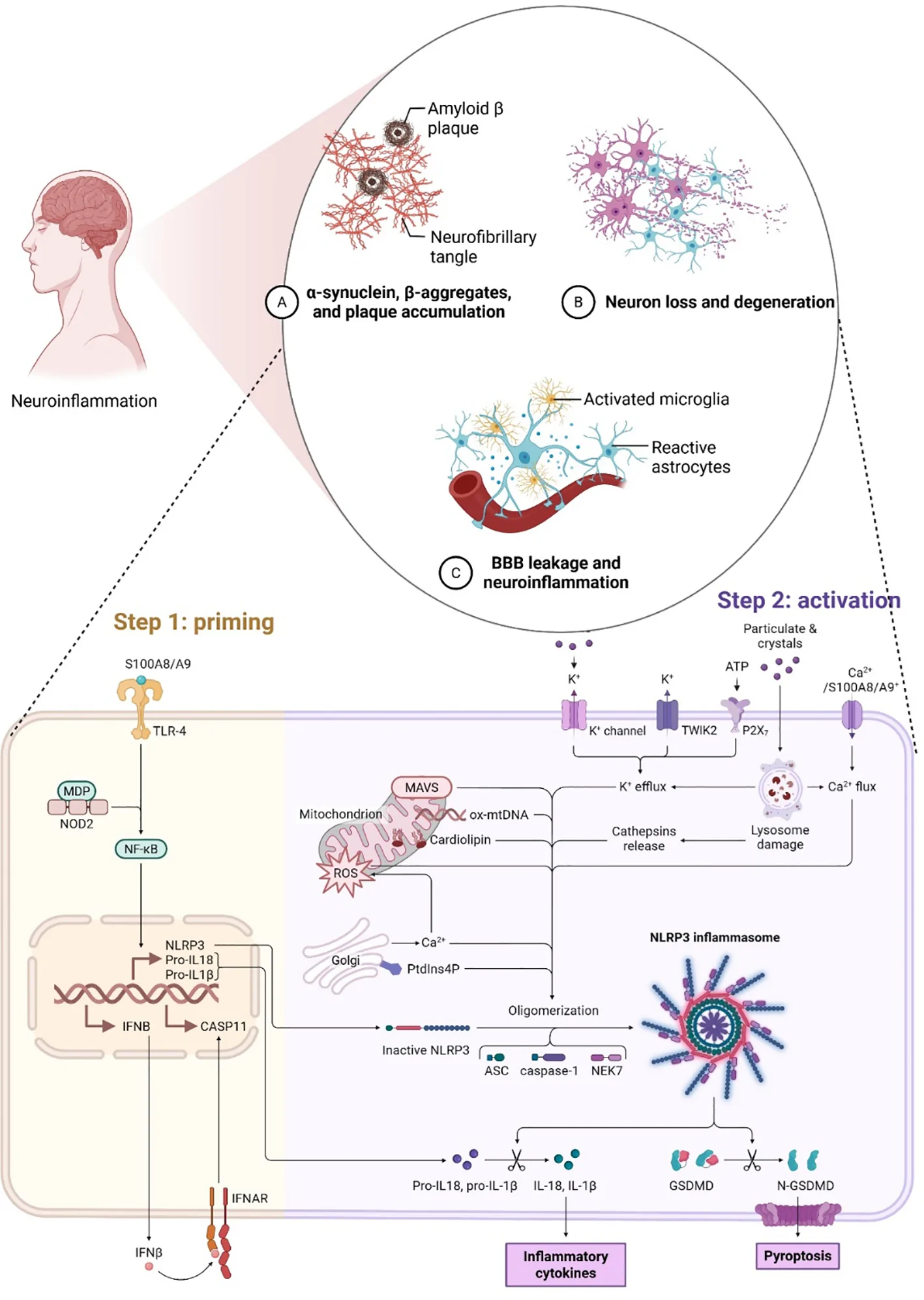
S100A8/A9-driven neuroinflammation through NLRP3 inflammasome activation. The upper panel illustrates major pathological drivers of neuroinflammation. Accumulation of alpha-synuclein, beta-amyloid plaques, and neurofibrillary tangles initiates chronic inflammatory signaling **(A)**, leading to progressive neuronal dysfunction and cell loss **(B)**. Blood-brain barrier leakage permits entry of inflammatory mediators and promotes activation of microglia and reactive astrocytes **(C)**. Microglia are activated in part through the release of S100A8 and S100A9 proteins from stressed or damaged cells, which engage TLR4 and induce NF-κB-mediated transcription of NLRP3, pro-IL-1β, and pro-IL-18. The lower panel depicts the two-step process of NLRP3 inflammasome activation. Priming occurs through TLR4 and NOD2 signaling, the IFN-β and CASP11 pathways, and NF-κB-driven upregulation of inflammasome components. Activation is triggered by potassium efflux, ATP-mediated P2X7 stimulation, calcium influx, particulate-induced lysosomal damage, mitochondrial reactive oxygen species, oxidized mitochondrial DNA, cardiolipin exposure, MAVS, and Golgi-derived PtdIns4P. These signals promote NLRP3 oligomerization and assembly with ASC, caspase-1, and NEK7, resulting in the maturation of IL-1β and IL-18, the release of inflammatory cytokines, and gasdermin D-dependent pyroptosis.

The NLRP3 inflammasome represents another major inflammatory pathway regulated by S100A8/A9. Activation of TLR4 and NF-κB by S100A8/A9 provides the priming signal required for NLRP3 expression, whereas ROS generation, potassium efflux, and TXNIP dissociation facilitate assembly of the inflammasome complex (2, 49). Activated NLRP3 subsequently promotes caspase-1 activation and maturation of IL-1β and IL-18, thereby amplifying inflammatory responses (43, 50, 51). S100A8/A9-mediated activation of the TLR4–NLRP3 signaling axis contributes to inflammation in numerous diseases, including contrast-induced acute kidney injury, acute lung injury, intervertebral disc degeneration, and subarachnoid hemorrhage (52–55) Within the CNS, S100A9-induced activation of NLRP3 in microglia exacerbates neuroinflammation, neuronal apoptosis, and early brain injury, whereas genetic deletion of S100A9 significantly improves neurological outcomes (53). These findings establish S100A8/A9 as an upstream regulator of inflammasome activation and a promising therapeutic target for neuroinflammatory disorders (**Figure 2**).

S100A8/A9 also activates the MAPK signaling pathway downstream of TLR4. Activation of ERK1/2, JNK, and p38 MAPK stimulates AP-1 transcription factors, leading to increased production of inflammatory cytokines and chemokines, including IL-6, IL-8, and TNF-α (30, 46). In neuronal tissues, S100A8 is markedly upregulated under hypoxic conditions and activates ERK/JNK signaling in neurons and microglia, promoting cyclooxygenase-2 (COX-2) expression, neuronal apoptosis, and neuroinflammation (33). Similarly, activation of MAPK signaling by S100A8/A9 contributes to oxidative stress, cognitive decline, and disease progression in Alzheimer’s disease, Parkinson’s disease, and spinal cord injury (33, 56). Thus, S100A8/A9-mediated MAPK activation represents a critical mechanism linking inflammatory signaling with neurodegeneration.

In addition to TLR4, S100A8/A9 signals through the RAGE, which serves as a major receptor for extracellular S100 proteins (57, 58). Engagement of RAGE activates multiple downstream pathways, including NF-κB, MAPK, and JAK2/STAT3 signaling, thereby sustaining inflammatory responses. Hyperoxidized S100A9 interacts with RAGE to activate PKCβ/ERK/JNK signaling, resulting in increased apoptosis, disruption of the OPG/RANKL balance, and tissue destruction in periodontal disease (59). Similarly, S100A8/A9 promotes fibrosis in intrauterine adhesion through activation of the RAGE–JAK2–STAT3 pathway, highlighting the broad pathological significance of this receptor beyond neurological disorders (60). Persistent activation of RAGE signaling by S100A8/A9 is therefore likely to contribute to chronic neuroinflammation through sustained activation of inflammatory transcriptional programs.

Reactive oxygen species (ROS) function as important secondary messengers downstream of S100A8/A9 signaling. Following TLR4 activation, S100A8/A9 stimulates NOX2-dependent ROS generation, which further activates NF-κB and promotes NLRP3 inflammasome assembly (2, 30, 49). ROS-mediated signaling amplifies cytokine production while simultaneously inducing mitochondrial dysfunction and neuronal apoptosis. Within the CNS, excessive ROS production contributes to oxidative stress, chronic microglial activation, and progressive neurodegeneration. Consequently, ROS-dependent signaling represents an important mechanism through which S100A8/A9 links peripheral inflammation to neuroinflammation and cognitive decline (33).

Collectively, these findings establish S100A8/A9 as a master regulator of inflammatory signaling whose inhibition may simultaneously suppress multiple pathogenic pathways responsible for chronic inflammation, neuroinflammation, and neurodegeneration (**Table 2**).

**Table 2:** Role of S100A8/A9 in NLRP3 inflammasome activation and related inflammatory conditions.

Mechanistic category	Experimental model/context	Role of S100A8/A9	Key evidence relevant to neuroinflammation, microglial activation, and the oral–brain axis	Therapeutic implication	References
Neuroinflammation	CNS neuroinflammatory conditions	Activates microglia and immune cells; promotes pro-inflammatory cytokine production	Elevated S100A8/A9 increases TNF-α, IL-1β, IL-6, and chemokine production, driving neuroinflammation in the CNS.	Inhibition of S100A8/A9 attenuates neuroinflammation.	(6)
	Hypoxia/ischemic injury models	Enhances inflammasome activation and cytokine release	S100A8/A9 upregulation exacerbates neuroinflammation and neuronal damage through IL-1β and IL-18 production.	Targeting S100A8/A9 or NLRP3 reduces neuronal injury.	(47)
Microglial Activation	Microglia *in vitro* (BV2/primary microglia)	Drives M1 microglial polarization and activation	S100A8/A9 increases iNOS, TNF-α, and IL-6 expression while suppressing anti-inflammatory (M2) markers.	Inhibition of S100A8/A9 or NF-κB suppresses microglial activation.	(48)
	Neuroinflammatory models (e.g., LPS or sterile injury)	Promotes microglial activation and cytokine release	S100A8/A9 deficiency or inhibition reduces microglial activation and inflammatory cytokines, protecting against CNS inflammation.	Blocking the S100A8/A9 pathway reduces microglia-driven neuroinflammation.	(49)
	Neurodegeneration (Alzheimer’s disease models)	Enhances microglial activation and neuroinflammatory responses	S100A8/A9 promotes microglial activation, NLRP3 inflammasome activation, and exacerbates cognitive deficits.	S100A8/A9 inhibition attenuates neuroinflammation and cognitive impairment.	(61)
NLRP3 Inflammasome Activation	Neuroinflammatory and neurodegenerative models	Primes and activates the NLRP3 inflammasome	S100A8/A9 acts upstream to promote ROS-dependent NF-κB activation and NLRP3 inflammasome assembly, resulting in chronic neuroinflammation.	Dual targeting of S100A8/A9 and NLRP3 may provide greater therapeutic benefit.	(62)
Oral–Brain Axis	Periodontitis (oral inflammation)	Links oral inflammation to microglial activation in the CNS	Periodontal inflammation increases circulating S100A8/A9, which activates microglia through TLR4/NF-κB/NLRP3 signaling and promotes neuroinflammation.	Targeting S100A8/A9 may interrupt the oral–brain inflammatory axis and reduce neuroinflammation.	(63)
	Oral pathogen/LPS exposure	Enhances microglial activation and cytokine release	Oral pathogens or LPS elevate S100A8/A9, aggravating microglial activation and neuroinflammatory responses.	Blocking S100A8/A9 or its receptors reduces oral pathogen-induced neuroinflammation.	(64)

### S100A8/A9-targeted natural and pharmacological inhibitors

S100A8/A9 amplifies neuroinflammation after traumatic brain injury by promoting neutrophil extracellular trap (NET) formation through ROS and PAD4 activation. Inhibition of S100A8/A9 reduces NET-mediated neuroinflammation and neuronal death, highlighting its potential as a therapeutic target (65). Bioinformatics and experimental analyses revealed that Cryptotanshinone, the active component of Qu-Shi-Xie-Zhuo decoction, binds to S100A8/A9 and inhibits the TLR4-NLRP3-IL-1β signaling pathway. This action reduces inflammatory cytokine production and immune cell infiltration, highlighting its potential as a therapeutic strategy for inflammatory disorders (66). Ponatinib induces cardiotoxicity by activating the S100A8/A9-TLR4-NLRP3-IL-1β signaling pathway in cardiac and myeloid cells, driving excessive myocardial and systemic inflammation. Targeting S100A8/A9 or NLRP3 effectively mitigates inflammation and cardiac dysfunction, highlighting potential therapeutic interventions (61). S100A9 is markedly elevated during LPS-driven lung injury in sepsis, and its inhibition reduces inflammation, apoptosis, and overall tissue damage. These protective effects occur through suppression of NLRP3 signaling, indicating S100A9 blockade as a promising therapeutic approach for septic lung injury (67). Metformin protects against sepsis-induced acute lung injury in young mice by suppressing the S100A8/A9-NLRP3-IL-1β axis and inhibiting NF-κB–mediated inflammation and pyroptosis. This intervention improves survival, reduces lung tissue damage, and lowers the expression of pro-inflammatory cytokines (68). Elevated S100A8 and S100A9 levels strongly correlate with septic left ventricular dysfunction, and experimental studies show that these alarmins directly drive inflammation and myocardial injury. Blocking S100A8/A9 with ABR-238901 or using S100A9-deficient mice effectively prevents or reverses septic cardiomyopathy, highlighting this pathway as a promising therapeutic target (69). S100A8 promotes liver fibrosis by inducing NLRP3 inflammasome-dependent pyroptosis in Kupffer cells through TLR4/NF-κB signaling and ROS production, which activates hepatic stellate cells and collagen deposition. Circulating GSDMD (Gasdermin D) may serve as a predictive biomarker for fibrosis progression (70) (**Table 3**).

**Table 3:** Therapeutics targeting S100A8/A9 in NLRP3 inflammasome activation across diseases.

Disease category	Drugs/compounds	Target/mechanism	Disease/condition	References
Cardiovascular Diseases	ABR-238901	S100A8/A9 blockade	Sepsis-induced myocardial dysfunction; ischemia-reperfusion injury; atherosclerosis	(71)
	Statins (e.g., atorvastatin)	Downregulates S100A8/A9; inhibits NF-κB and NLRP3 activation	Atherosclerosis; myocardial infarction	(72)
Sepsis and Infectious Diseases	Metformin	Suppresses S100A8/A9-NLRP3-IL-1β axis; inhibits NF-κB-mediated inflammation and pyroptosis	Sepsis-induced acute lung injury; septic shock	(73)
	Dexamethasone	Reduces S100A8/A9 expression; suppresses NLRP3 inflammasome	Sepsis; systemic inflammatory response syndrome	(74)
Inflammatory and Autoimmune Diseases	Cryptotanshinone	Binds S100A8/A9; inhibits TLR4-NLRP3-IL-1β signaling	Inflammatory disorders (Qu-Shi-Xie-Zhuo decoction); rheumatoid arthritis	(66)
	Resveratrol	Downregulates S100A8/A9; suppresses NLRP3 activation and IL-1β release	Rheumatoid arthritis; systemic lupus erythematosus	(75)
	Curcumin	Inhibits S100A8/A9-TLR4 interaction; blocks NLRP3 inflammasome	Rheumatoid arthritis; inflammatory bowel disease	(76)
Fibrosis and Gynecological Diseases	Menstrual blood-derived stromal cells (MenSCs)	Reduces S100A8/A9 expression; mitigates fibrosis	Intrauterine adhesion	(77)
	Pirfenidone	Inhibits S100A8/A9-mediated NLRP3 activation; attenuates fibrosis	Pulmonary fibrosis; liver fibrosis; renal fibrosis	(2)
	Galunisertib (LY2157299)	Suppresses S100A8/A9-NLRP3-IL-1β axis	Liver fibrosis; systemic sclerosis	(78)
Neurological and Other Diseases	Minocycline	Decreases S100A8/A9; inhibits microglial NLRP3 activation	Alzheimer’s disease; stroke; traumatic brain injury	(79)
	Oxaliplatin (adjunct targeting S100A8/A9)	Reduces S100A8/A9 release from neutrophils; inhibits NLRP3 inflammasome	Diabetic nephropathy	(80)

### S100A8/A9 in oral inflammation and neuroimmune signaling

Oral inflammation is a localized immune response triggered by microbial dysbiosis, periodontal pathogens, mucosal injury, and other environmental or systemic factors (81). Among the mediators involved in this process, S100A8 and S100A9 play a central role in orchestrating innate immune responses. As DAMP molecules, S100A8/A9 are rapidly released at sites of inflammation, where they contribute to antimicrobial defense, leukocyte recruitment, and amplification of inflammatory signaling (82). Because of their abundance and responsiveness to inflammatory stimuli, S100A8/A9 levels closely reflect disease progression and tissue inflammatory burden. In periodontal diseases, S100A8/A9 expression increases with disease severity. Gingivitis, a reversible plaque-induced inflammatory condition, is associated with a modest 2-5-fold elevation of S100A8/A9 levels in gingival crevicular fluid (GCF), reflecting the early influx of neutrophils in response to microbial challenge (12, 13). In contrast, stage II/III periodontitis exhibits a substantially greater increase (10–50-fold), correlating with periodontal pocket depth, attachment loss, persistent neutrophilic inflammation, and osteoclast-mediated tissue destruction (83). Similar elevations have been reported in peri-implantitis, where dysbiotic biofilms formed on implant surfaces stimulate robust inflammatory responses. The stability, abundance, and strong association of salivary and GCF S100A8/A9 levels with disease severity have established S100A8/A9 as a promising non-invasive biomarker for the diagnosis, staging, and monitoring of periodontal diseases (84).

## Cellular sources of S100A8/A9 in gingivitis, periodontitis, and peri-implantitis

The cellular origin of S100A8/A9 within the oral cavity evolves with disease progression. During the early stages of gingival inflammation, neutrophils serve as the predominant source of S100A8/A9. S100A8/A9 constitutes up to 40–60% of the soluble cytoplasmic protein content of neutrophils and is released during diapedesis, degranulation, and neutrophil extracellular trap (NET) formation in response to oral dysbiosis (85, 86) Consequently, neutrophil-derived S100A8/A9 dominates the inflammatory milieu of gingivitis and early periodontal disease. Monocytes and macrophages become increasingly important contributors as inflammation progresses to chronic periodontitis. Secretion of inflammatory cytokines such as IFN-γ and TNF-α induces S100A8/A9 expression in these cells, which subsequently release the proteins during tissue remodeling and chronic inflammatory responses (86). Gingival epithelial cells, including junctional, sulcular, and oral epithelial subtypes, also contribute significantly. Although these cells constitutively express low levels of S100A8/A9, microbial stimuli such as *Porphyromonas gingivalis* lipopolysaccharide and *Fusobacterium nucleatum* markedly enhance their expression and secretion, thereby amplifying local innate immune responses. Additional sources include fibroblasts, endothelial cells, activated Th17 cells, and mast cells, which collectively sustain inflammatory signaling within periodontal tissues (14, 87–90).

## S100A8/A9 as a mediator of the oral–brain axis

Although S100A8/A9 is produced locally within inflamed periodontal tissues, increasing evidence suggests that its biological effects extend beyond the oral cavity. Chronic periodontal inflammation is associated with sustained release of S100A8/A9 into the gingival crevicular fluid, saliva, and systemic circulation, where these proteins function as potent circulating DAMPs capable of amplifying inflammatory responses in distant organs. Elevated systemic S100A8/A9 promotes peripheral immune activation by stimulating monocytes, neutrophils, and endothelial cells through TLR4 and RAGE signaling, leading to increased production of pro-inflammatory cytokines including TNF-α, IL-1β, IL-6, and IL-17 (14, 30, 46). In parallel, systemic inflammation induced by chronic periodontal disease can compromise blood-brain barrier (BBB) integrity by disrupting endothelial tight junctions and increasing vascular permeability, thereby facilitating the entry of circulating inflammatory mediators and immune cells into the CNS. Although direct translocation of S100A8/A9 across the BBB remains to be fully established, peripheral S100A8/A9 may indirectly influence the CNS by promoting cytokine-mediated endothelial activation, enhancing leukocyte trafficking, and priming brain-resident immune cells. These mechanisms provide a plausible molecular framework through which persistent oral inflammation may communicate with the brain, positioning S100A8/A9 as a key mediator linking periodontal inflammation to neuroimmune dysregulation and chronic neuroinflammation.

## Microglial activation and neuroimmune signaling

Microglia are the resident innate immune cells of the CNS and play a critical role in maintaining neural homeostasis through debris clearance, synaptic remodeling, and immune surveillance (91, 92). In response to infectious, inflammatory, or degenerative stimuli, microglia undergo activation characterized by morphological transformation, enhanced phagocytic activity, and the production of pro-inflammatory mediators, including IL-1β, TNF-α, IL-6, IL-12, IL-23, CCL2, CCL3, CCL4, CCL5, CXCL8, and reactive oxygen species (93). While transient activation is essential for host defense and tissue repair, persistent microglial activation can drive chronic neuroinflammation and contribute to neuronal dysfunction and neurodegenerative disease progression (94). Accumulating evidence identifies S100 family proteins, particularly S100A8 and S100A9, as important regulators of microglial activation and neuroimmune signaling. Extracellular S100A8/A9 engages pattern-recognition receptors such as TLR4 and the receptor for advanced glycation end products (RAGE), triggering ERK/JNK-dependent NF-κB signaling and subsequent production of pro-inflammatory cytokines including TNF-α and IL-6 (14). Experimental studies have demonstrated that S100A8/A9 promotes polarization of microglia toward a pro-inflammatory M1 phenotype, thereby amplifying neuroinflammatory responses and tissue injury (15). Similarly, S100A8 can activate ERK/JNK and NLRP3 signaling pathways in microglia, leading to increased cytokine production, cyclooxygenase-2 expression, and neuronal apoptosis (33). Elevated S100A8/A9 expression has also been implicated in several neurological disorders, including Alzheimer’s disease, multiple sclerosis, sepsis-associated encephalopathy, traumatic brain injury, glioma, and postoperative cognitive dysfunction, underscoring its broader significance in neuroimmune regulation (31, 95).

## Linking oral inflammation to microglial activation through S100A8/A9

**Figure 3:**
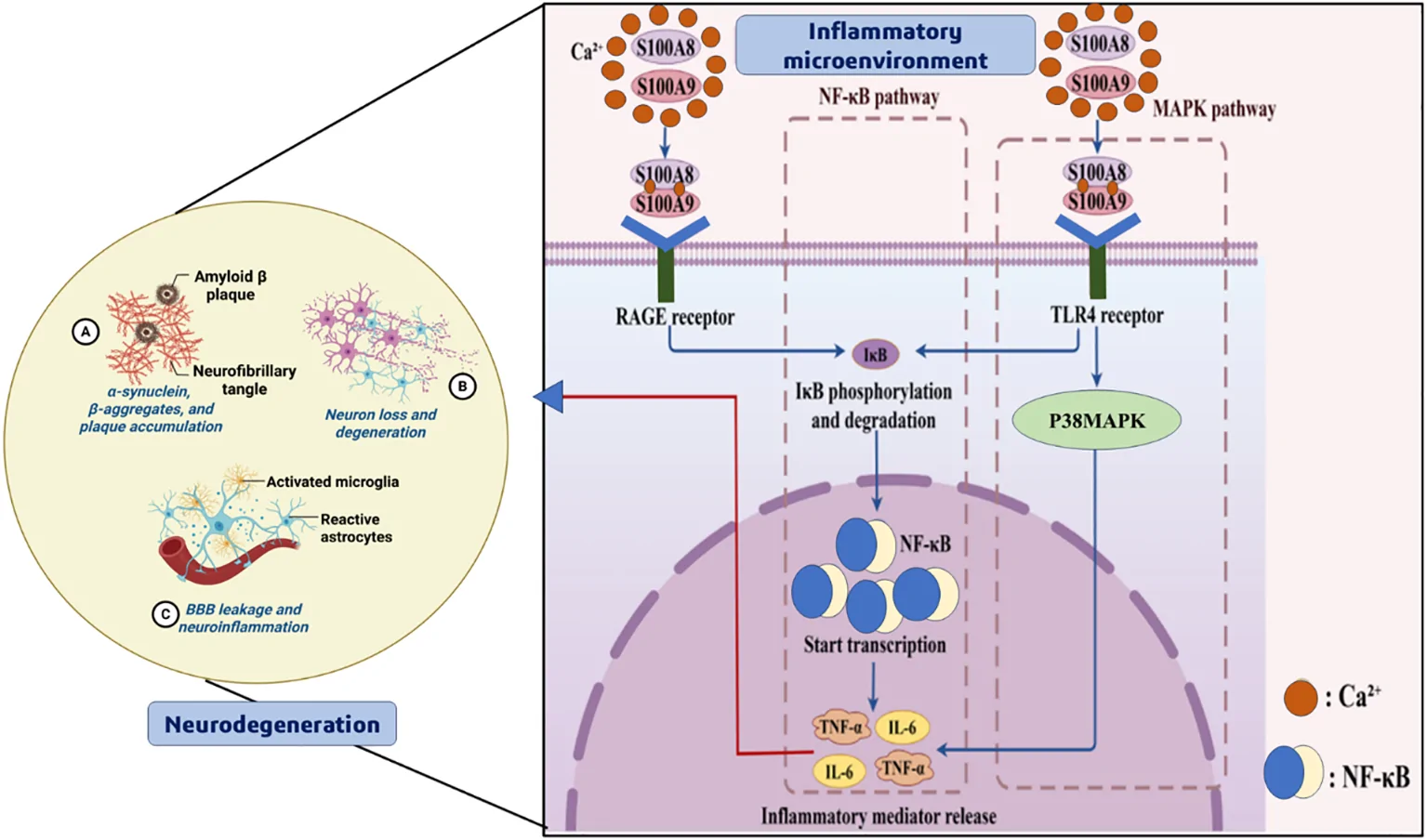
Schematic illustration of S100A8/A9-mediated neuroinflammatory signaling pathways. The figure depicts the role of S100A8/A9 in neuroinflammation and neurodegeneration. In hippocampal neurons, S100A8/A9 interacts with RAGE and TLR4 receptors to initiate intracellular signaling cascades. Activation of the NF-κB pathway involves phosphorylation and degradation of IκB, which permits NF-κB translocation into the nucleus and subsequent transcription of pro-inflammatory genes. In parallel, TLR4 activation triggers the MAPK pathway, leading to p38-MAPK phosphorylation. Together, these signaling events promote the release of inflammatory mediators such as TNF-α and IL-6, thereby amplifying neuroinflammation. In addition, neuroinflammation is associated with amyloid-β plaque accumulation, neurofibrillary tangle formation, α-synuclein/β-aggregates deposition, neuron loss and degeneration, as well as activation of microglia, reactive astrocytes, Blood Brain Barrier(BBB) leakage, and progression towards neurodegeneration. Created with BioRender.com.

The emerging concept of the oral–brain axis suggests that chronic oral inflammation may influence neuroinflammatory processes through systemic dissemination of inflammatory mediators. Given their high expression in periodontal tissues and potent immunomodulatory properties, S100A8/A9 proteins are attractive candidates linking oral inflammation to microglial activation. Periodontal inflammation is characterized by sustained release of S100A8/A9 from neutrophils, macrophages, and epithelial cells into local tissues and systemic circulation. Once elevated systemically, these DAMP molecules can engage TLR4 and RAGE signaling pathways that are also highly expressed on microglia, thereby promoting neuroimmune activation (96) (**Figure 3**).

Support for this mechanism comes from studies showing that peripheral inflammatory insults induce S100A8/A9 expression in circulating leukocytes and CNS-associated immune cells. For example, carrageenan-induced peripheral inflammation increases S100A8 and S100A9 expression in neutrophils associated with spinal cord vasculature, suggesting a pathway through which peripheral inflammatory signals can be communicated to the CNS (39). Furthermore, S100A8-driven activation of the TLR4–MyD88 axis has been shown to exacerbate neuroinflammation in models of traumatic brain injury and postoperative cognitive dysfunction, while blockade of S100A8 or TLR4 signaling attenuates microgliosis and improves neurological outcomes (53). Given that chronic periodontal diseases represent a persistent source of circulating S100A8/A9, it is plausible that these proteins contribute to systemic inflammatory priming of microglia and subsequent neuroinflammatory responses. Although direct mechanistic evidence linking periodontal-derived S100A8/A9 to microglial activation remains limited, the shared inflammatory pathways involving TLR 4, RAGE, NF-κB, and cytokine signaling strongly support a functional connection between oral inflammation and CNS immune dysregulation.

## S100A8/A9 as a biomarker

S100A8 and S100A9 are consistently upregulated in a broad spectrum of inflammatory disorders, cancers, and immune-mediated diseases. Their expression is closely associated with neutrophil activation and inflammatory signaling, making them attractive diagnostic and prognostic biomarkers. Elevated S100A8/A9 levels have been reported in several systemic pathologies, including ischemic stroke, inflammatory bowel disease, and adamantinomatous craniopharyngioma, where their expression correlates with disease severity, inflammatory burden, and clinical outcomes (97–99). The diagnostic potential of S100A8/A9 is particularly evident in oral diseases. For instance, S100A8 is significantly upregulated in symptomatic irreversible pulpitis suggesting its role in pulpal inflammation and disease progression (100). Similarly, numerous studies have reported elevated salivary and gingival crevicular fluid levels of S100A8 and S100A9 in periodontitis. Their expression correlates with key clinical parameters, including bleeding on probing, pocket depth, and disease severity, while also responding to periodontal therapy (12, 63). Proteomic analyses have further identified S100A8/A9 among the most significantly upregulated salivary proteins in periodontitis, supporting their utility as non-invasive biomarkers for disease screening and monitoring (101). In addition to their biomarker potential, experimental studies indicate that S100A8/A9 contributes to periodontal homeostasis by regulating microbial composition and limiting inflammatory tissue damage (5).

Among oral pathologies, the strongest evidence for the clinical application of S100A8/A9 has emerged from oral squamous cell carcinoma (OSCC). Proteomic profiling of oral brush biopsies demonstrated that S100A8 and S100A9 can effectively discriminate between normal, inflammatory, and malignant oral lesions with high sensitivity and specificity (102). Likewise, elevated salivary S100A8 levels have been shown to correlate with tumor stage and disease progression, highlighting their value for non-invasive diagnosis and disease monitoring (95).

Thus, the consistent overexpression of S100A8/A9 across oral inflammatory conditions and oral malignancies, together with their detectability in saliva and other accessible biofluids, underscores their potential as clinically relevant biomarkers for disease diagnosis, prognosis, and therapeutic monitoring.

## Pharmacological targeting of S100A8/A9

S100A8/A9, a central mediator of the host inflammatory response, represents an attractive therapeutic target at multiple levels, including its expression, activity, and downstream signaling. S100A8/A9 and its effector functions can be targeted at upstream modulators that suppress S100A8/A9 gene expression or secretion.

Therapeutic targeting of S100A8/A9 can be broadly divided into direct and indirect strategies based on whether the intervention acts on the S100A8/A9 complex itself or on the signaling pathways that regulate its activity and expression (103). Direct treatment approaches seek to target S100A8/A9 itself, by blocking ligand function, inhibiting complex formation, or neutralizing the protein complex before it interacts with downstream receptors. Since these methods operate at the level of the alarmin rather than its signaling network, they differ conceptually from pathway-level inhibition (104). Examples of direct targeting include anti-S100A8/A9 neutralizing monoclonal antibodies such as Ab45, which suppressed S100A8/A9-mediated melanoma mobility and lung metastasis in mice (103). For heterodimer-focused targeting, the anti-S100A8/A9 antibody described in preclinical studies is especially relevant because it targets the S100A8/A9 complex rather than only one subunit.

**Figure 4:**
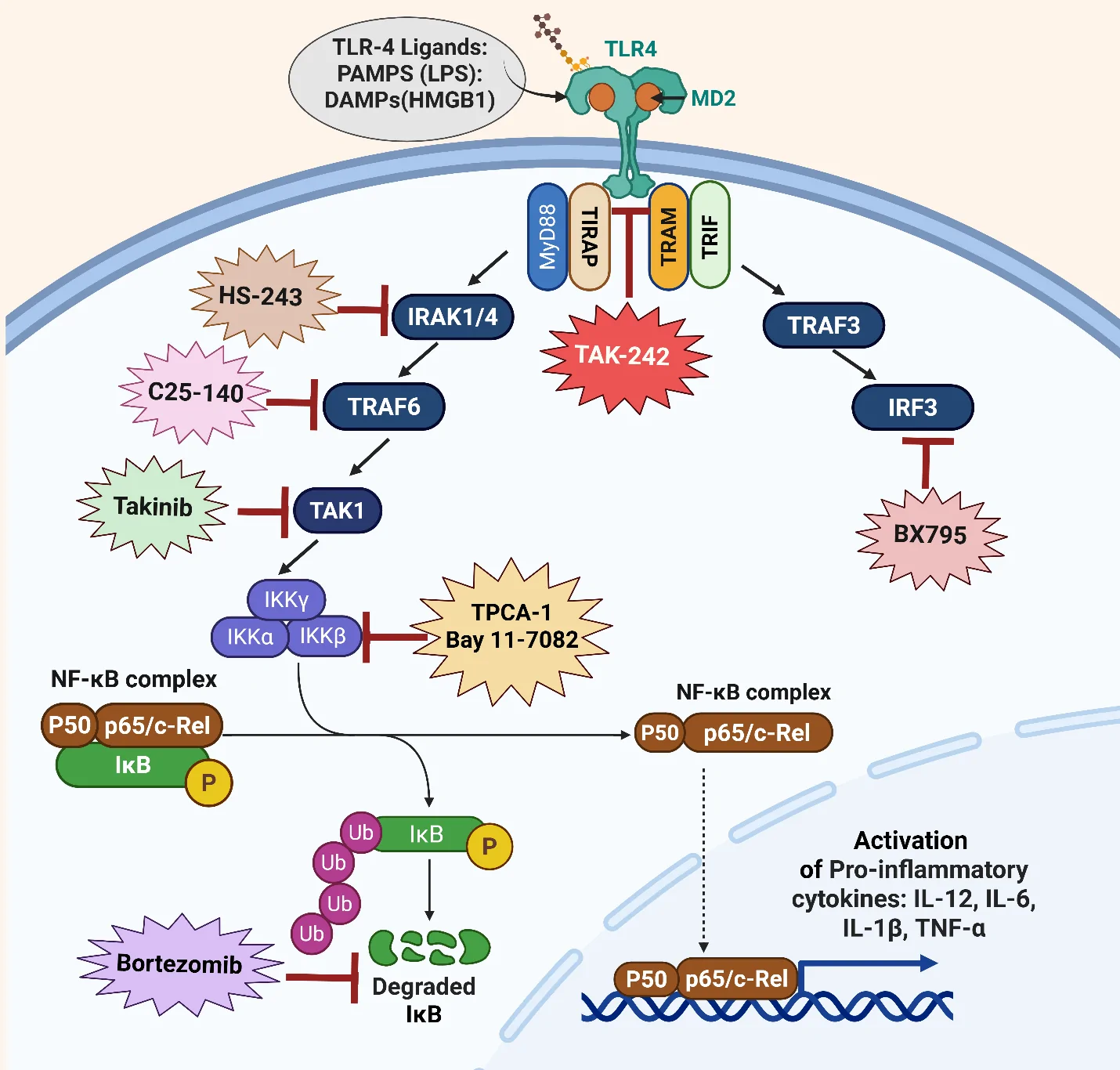
NF-κB–mediated regulation of S100A8/A9 expression and pharmacological inhibition of S100A8/A9-associated inflammation. TLR4 activation by PAMPs (pathogen-associated molecular patterns) such as LPS and DAMPs (damage-associated molecular patterns) like High Mobility Group Box 1 (HMGB1) initiates downstream signaling through MyD88/TIR domain-containing adaptor protein (TIRAP) and TIR-domain-containing adapter molecule 1(TRAM)/TIR domain-containing adaptor-inducing interferon-β (TRIF) adaptor pathways. HS-243 inhibits IRAK1/4, C25–140 targets the TRAF6-Ubc13 interaction, and Takinib inhibits TAK1 in the MyD88-dependent branch, which suppresses NF-κB signaling and IKK complex activation. IKK-mediated NF-κB activation is inhibited by TPCA-1 and Bay 11-7082, whereas proteasome-dependent IκB degradation is blocked by bortezomib. In the TRIF-dependent branch, BX795 prevents IRF3 activation downstream of TLR4 signaling, while TAK-242 (resatorvid) which is a selective TLR4 antagonist, covalently binds to the intracellular Toll/interleukin-1 receptor (TIR) domain of TLR4. This binding prevents the recruitment of the MyD88-dependent signaling complex by interfering with the interaction between the TLR4 TIR domain and the adaptor protein TIRAP (Mal). As a result, pro-inflammatory cytokines including TNF-α, IL-6, and IL-1β are inhibited by blocking downstream activation of NF-κB. TRAF3 inhibitor remains in the discovery line, and no established direct TRAF3 inhibitor is currently available for routine use. Created with BioRender.com.

Indirect strategies target upstream receptors or downstream signaling pathways, such as TLR4, RAGE, or NF-κB, to reduce S100A8/A9-driven inflammatory responses (105). Among upstream pathways, the NF-κB signaling axis, an essential transcriptional regulator of S100A8 and S100A9 in innate immune cells. Small-molecule inhibitors like TPCA-1, Bay 11-7082, and BMS-345541 considerably lower S100A8/A9 transcription in neutrophil and monocyte cell lines as well as in murine colitis models, by preventing the IKKβ-dependent activation of the conventional NF-κB pathway, IKKβ facilitates the degradation of IκB in inflammatory conditions, allowing NF-κB to translocate into the nucleus and trigger the transcription of pro-inflammatory genes, such as S100A8 and S100A9. This signaling cascade is disrupted, NF-κB activation is decreased, and S100A8/A9 expression decreases as a result of IKKβ inhibition (5) (**Figure 4**).

Similarly, TAK-242 (resatorvid) mainly alters the TLR4-driven innate immune signaling network. TLR4 recognizes pathogen-associated molecular patterns (PAMPs), such as lipopolysaccharide (LPS), and damage-associated molecular patterns (DAMPs), such as high-mobility group box 1 (HMGB1), with help of co-receptors such as MD-2 (myeloid differentiation factor 2), Ly86 (lymphocyte antigen 86), CD14 (cluster of differentiation 14), and CD180 (cluster of differentiation 180). Upon activation, TLR4 signals through two major pathways: the MyD88-dependent pathway, involving TIRAP/Mal (TIR domain-containing adaptor protein/MyD88-adapter-like), MyD88 (myeloid differentiation primary response 88), IRAK1/4 (interleukin-1 receptor-associated kinase 1/4), TRAF6 (TNF receptor-associated factor 6), IKK (IκB kinase), and NF-κB (nuclear factor kappa B), leading to production of pro-inflammatory cytokines. and the TRIF-dependent pathway, involving TRAM (TRIF-related adaptor molecule), TRIF (TIR-domain-containing adapter-inducing interferon-β), TRAF3 (TNF receptor-associated factor 3), TBK1 (TANK-binding kinase 1), and IRF3 (interferon regulatory factor 3), resulting in IFN-β (interferon beta) and ISRE (interferon-stimulated response element) activation (**Figure 4**). Thus, TAK-242 inhibits downstream inflammatory and interferon-mediated signaling by binding to the intracellular TIR domain of TLR4 and interfering with adaptor recruitment (106).

Among direct S100A8/A9 inhibitors, quinoline-3-carboxamide derivatives such as paquinimod (ABR-215757), tasquinimod (ABR-215050), and ABR-238901 exhibit high-affinity binding to S100A9 (107–110). These compounds disrupt the S100A9–TLR4 interaction and inhibit activation of downstream proinflammatory signaling in myeloid and tumor-infiltrating immune cells and prevent vascular calcification in patients with chronic kidney disease (111). Notably, paquinimod exhibited high efficacy in Phase II clinical trials for systemic lupus erythematosus and systemic sclerosis, reducing circulating S100A8/A9 levels and alleviating inflammatory pathology without inducing overt immunosuppression (112). Lycorine, an alkaloid from hot water extracts of *Crinum asiaticum*, inhibited induction of apoptosis by S100A8/A9 (71). Lycoricinidol, a similar natural compound, inhibited S100A8/A9 -induced cytotoxicity at more than a 10-fold lower concentration than lycorine (113). Anti-RAGE antibodies and soluble RAGE (sRAGE) decoy receptors sequester S100A8/A9 and prevent its interaction with membrane-bound RAGE. As a result, they interrupt autocrine amplification loops (72). Owing to its multifaceted involvement in diverse pathological conditions, S100A8/A9 functions not only as a biomarker of inflammation but also as a compelling therapeutic target. The availability of high-resolution structural data has revealed multiple allosteric sites such as the dimer interface or the sites that are involved in receptor binding, providing a framework for the rational design of next-generation inhibitors. Exploiting these sites could facilitate the discovery of compounds that modulate S100A8/A9 effector functions via diverse mechanisms. Notably, its secretory nature and intrinsic antimicrobial activity implicate that recombinant S100A8/A9, or its metal-chelating domains, could serve as antimicrobial agents for localized applications such as ectopic injuries. However, such approaches would require careful engineering to attenuate its immunostimulatory properties. In parallel, high-affinity peptides developed against S100A8/A9 represent an additional promising strategy; although initially designed for diagnostic applications, these molecules may be further optimized to function as specific inhibitors of S100A8/A9 activity (114).

Targeting S100-associated inflammatory signaling has yielded several candidate interventions. **Table 4** shows that blockade of TLR4, inhibition of IKKβ, decoy sequestration of RAGE ligands, and cytokine-based immunoregulatory approaches can reduce S100A8/A9 -associated inflammation and improve disease outcomes in preclinical and translational settings. However, the therapeutic utility of these approaches remains limited by pathway specificity, host-defense concerns, and delivery challenges (14).

**Table 4:** Overview of S100A8/A9-targeted therapeutics: experimental models, clinical stage, and translational challenges.

Drug	Target/Mechanism	Disease area	Highest clinical stage	Preclinical findings	Clinical findings	Limitations/Status	References
Tasquinimod (ABR-215050)	S100A9 inhibitor; HDAC4 allosteric modulator	mCRPC, MM	Phase III (discontinued in PC)	Reduced tumor burden and MDSC inhibition in murine prostate cancer models	Phase II: Improved PFS (7.6 vs. 3.3 mo; P = 0.0042); Phase III (10TASQ10, N = 1,245): Prolonged PFS but no OS benefit; development discontinued	Mechanism ambiguity (dual S100A9/HDAC4 binding); lack of OS benefit	(115, 116)
Paquinimod (ABR-215757)	S100A9 inhibitor; blocks TLR4/RAGE binding	SLE, SSc, IPF (preclinical)	Phase II (per PubChem)	Reduced fibrosis in murine BLM-lung fibrosis; ameliorated Tsk-1 skin fibrosis by M2→M1 macrophage polarization	Phase Ib: Well-tolerated up to 3 mg/day; linear PK in SLE	Limited published efficacy data; no Phase III trials yet	(112, 117, 118)
Neutralizing antibodies (e.g., Ab45)	S100A8/A9 heterodimer	Cancer (melanoma)	Preclinical only	Blocked melanoma lung metastasis in mice	No human clinical trials	Lack of clinical development data	(103)
Natural products (kaempferol, glycyrrhizin)	S100A8/A9 inhibition	Various inflammatory diseases	Preclinical only	Anti-inflammatory effects in cell/animal models	No clinical trials	Lack of specificity; bioavailability concerns	(119)

### Translational challenges and clinical development status of S100A8/A9-targeted therapies

A critical evaluation of the translational potential of S100A8/A9-targeted therapies reveals a substantial gap between preclinical promise and clinical validation. The majority of evidence derives from *in vitro* studies and animal models. For instance, the S100A9 inhibitor paquinimod reduced skin fibrosis in the tight skin 1 mouse model of systemic sclerosis by polarizing macrophages from M2 to M1 phenotype (117), and ameliorated bleomycin-induced pulmonary fibrosis by suppressing endothelial-mesenchymal transition and reducing neutrophil infiltration (118). Similarly, tasquinimod (ABR-215050), an oral quinoline-3-carboxamide that binds S100A9 and HDAC4, demonstrated robust anti-tumor efficacy across multiple human prostate cancer xenograft models (115, 116) and suppressed myeloid-derived suppressor cell function in preclinical models (116).

However, translation to human clinical benefit has been limited. Tasquinimod advanced to Phase III evaluation in metastatic castration-resistant prostate cancer (mCRPC) in the 10TASQ10 trial, which enrolled 1,245 men (120). While a Phase II trial had shown improved progression-free survival (PFS: 7.6 vs. 3.3 months; P = 0.0042) (116).The Phase III trial, according to the developer, showed that tasquinimod prolonged progression-free survival (PFS) but did not extend overall survival, leading to discontinuation of prostate cancer development. Paquinimod (ABR-215757) has completed Phase Ib evaluation in systemic lupus erythematosus, where doses up to 3.0 mg/day were well tolerated with linear pharmacokinetics suitable for once-daily dosing (112), and has shown promising effects in experimental lupus models comparable to prednisolone (112).

Several translational challenges impede clinical success. First, S100A8/A9 proteins are integral to normal innate immune responses, and their complete suppression may compromise host immunity (121, 122). Second, the mechanism of action of quinoline-3-carboxamides remains incompletely understood. Tasquinimod, for example, also binds HDAC4 with high affinity, complicating attribution of clinical effects solely to S100A9 inhibition (115). Third, no S100A8/A9-targeted monoclonal antibody has entered clinical trials to date, despite preclinical efficacy of neutralizing antibodies such as Ab45 in melanoma models (123). Fourth, the disconnect between PFS improvement and OS benefit observed with tasquinimod highlights the challenge of translating anti-inflammatory mechanisms into survival advantage in oncology.

In summary, while preclinical data strongly support S100A8/A9 as a therapeutic target across multiple disease models, clinical validation remains sparse (**Table 4**). Only tasquinimod has reached Phase III, where it failed to meet its primary survival endpoint despite showing anti-progression activity. No S100A8/A9-targeted biologic has advanced to human trials. Future development must address mechanism clarity, patient stratification, and the selection of clinically meaningful endpoints to bridge the translational barriers.

### Critical evaluation of S100A8/A9: beyond pro-inflammation-biomarker specificity, anti-inflammatory roles, and targeting risks

S100A8/A9 is not exclusively pro-inflammatory. Under specific conditions, it exhibits anti-inflammatory and homeostatic properties. S100A8-S-nitrosylation (S100A8-SNO) suppresses mast cell activation and leukocyte adhesion/extravasation in the microcirculation, representing a distinct anti-inflammatory mechanism (124). In the lung, S100A8/A9 and S100A9 reduce neutrophil influx in acute lung injury provoked by LPS challenge, with novel pathways including increased sirtuin-1 induction and STAT3 activation that may dampen NF-κB signaling (125). S100A8 also promotes IL-10 expression in airway epithelial cells, impairing LPS-induced neutrophil infiltration and reducing pro-inflammatory cytokine induction (126). Furthermore, S100A8/A9 can bind pro-inflammatory cytokines (IL-1β, IL-6, TNF-α) non-covalently, suggesting a capacity to trap and neutralize inflammatory mediators (127).

While S100A8/A9 offers neutrophil specificity and stability in biofluids, several limitations constrain its diagnostic utility. First, CRP remains more sensitive and specific for bacterial infections in systemic lupus erythematosus (AUC 0.966 vs. 0.732 for S100A8/A9 (128). Second, fecal calprotectin is physiologically elevated in infants younger than 4 years and shows labor-associated stress increases in neonates, necessitating age-adjusted reference ranges (129). Third, systemic inflammatory confounders-including obesity, diabetes, smoking, and chronic kidney disease-elevate baseline S100A8/A9 independently of the target disease (129). Fourth, assay platform variability complicates multicenter comparisons (130). Finally, the lack of tissue specificity-elevation across virtually all inflammatory conditions, limits diagnostic utility without clinical context.

## Summary and future perspectives

S100A8 and S100A9, as key alarmins of inflammation, are markedly elevated in nearly all inflammatory conditions. Although substantial evidence highlights the role of S100A8/A9 in inflammatory disease biology, the precise defense mechanisms of S100A8/A9 remain poorly understood. While numerous studies exist on S100A8 and S100A9 individually, detailed functional characterizations of the S100A8/A9 complex are limited (2).

Collectively, the evidence reviewed here underscores S100A8 and S100A9 as pivotal mediators of neuroinflammation, acting through microglial activation and downstream NF-κB signaling to drive the release of pro-inflammatory cytokines, including IL-6 and TNF-α. This signaling axis, further amplified by astrocytic involvement, contributes substantially to the pathological progression of Alzheimer’s disease, Parkinson’s disease, and related neurodegenerative disorders (131).

The convergence of S100A8/A9 at an upstream regulatory node of this inflammatory cascade positions them as compelling therapeutic targets. Although several drug candidates modulating these proteins have shown promise in preclinical and early-stage investigations, their translation into validated clinical therapies remains to be fully established. Rigorous target validation, improved selectivity of S100A8/A9-directed agents, and a deeper understanding of their crosstalk with other neuroinflammatory pathways, as well as more detailed functional characterization of the S100A8/A9 complex itself, should be the next essential steps.

A particularly promising future direction lies in the structural characterization of S100A8 and S100A9, including their calcium-binding EF-hand motifs and the interface residues mediating heterodimer (calprotectin) formation and receptor engagement with RAGE and TLR4. Identifying and targeting these specific structural motifs could enable the rational design of small-molecule or peptide-based inhibitors that selectively disrupt S100A8/A9-receptor interactions, offering a more precise, structure-guided therapeutic strategy for modulating neuroinflammation in neurodegenerative disease.

S100A8/A9-targeted intervention guided by both functional and structural insights holds the potential to shift the therapeutic paradigm from symptomatic management toward direct modulation of the inflammatory processes underlying neurodegeneration, representing a genuinely game-changing approach to the treatment of Alzheimer’s, Parkinson’s, and other neuroinflammatory-driven diseases.
